# Magnetically directed antioxidant and antimicrobial agent: synthesis and surface functionalization of magnetite with quercetin

**DOI:** 10.7717/peerj.7651

**Published:** 2019-11-20

**Authors:** Syed Tawab Shah, Wageeh A. Yehye, Zaira Zaman Chowdhury, Khanom Simarani

**Affiliations:** 1 Nanotechnology & Catalysis Research Centre (NANOCAT), Institute for Advanced Studies, University of Malaya, Kuala Lumpur, Malaysia; 2 Institute of Biological Sciences, Faculty of Science, University of Malaya, Kuala Lumpur, Malaysia

**Keywords:** Quercetin, Functional, Nanoantioxidant, Magnetite, DPPH, Combinatorial properties

## Abstract

Oxidative stress can be reduced substantially using nanoantioxidant materials by tuning its surface morphological features up to a greater extent. The physiochemical, biological and optical properties of the nanoantioxidants can be altered by controlling their size and shape. In view of that, an appropriate synthesis technique should be adopted with optimization of the process variables. Properties of magnetite nanoparticles (IONP) can be tailored to upgrade the performance of biomedicine. Present research deals with the functionalization IONP using a hydrophobic agent of quercetin (Q). The application of quercetin will control its size using both the functionalization method including in-situ and post-synthesis technique. In in-situ techniques, the functionalized magnetite nanoparticles (IONP@Q) have average particles size 6 nm which are smaller than the magnetite (IONP) without functionalization. After post functionalization technique, the average particle size of magnetite IONP@Q2 determined was 11 nm. The nanoparticles also showed high saturation magnetization of about 51–59 emu/g. Before starting the experimental lab work, Prediction Activity Spectra of Substances (PASS) software was used to have a preliminary idea about the biological activities of Q. The antioxidant activity was carried out using 2, 2-diphenyl-1-picrylhydrazyl (DPPH) assay. The antibacterial studies were carried out using well diffusion method. The results obtained were well supported by the simulated results. Furthermore, the values of the half maximal inhibitory concentration (IC50) of the DPPH antioxidant assay were decreased using the functionalized one and it exhibited a 2–3 fold decreasing tendency than the unfunctionalized IONP. This exhibited that the functionalization process can easily enhance the free radical scavenging properties of IONPs up to three times. MIC values confirms that functionalized IONP have excellent antibacterial properties against the strains used (*Staphylococcus aureus, Bacillus subtilis and Escherichia coli*) and fungal strains (*Aspergillus niger*, *Candida albicans*, *Trichoderma* sp. and *Saccharomyces cerevisiae*). The findings of this research showed that the synthesized nanocomposite has combinatorial properties (magnetic, antioxidant and antimicrobial) which can be considered as a promising candidate for biomedical applications. It can be successfully used for the development of biomedicines which can be subsequently applied as antioxidant, anti-inflammatory, antimicrobial and anticancer agents.

## Introduction

The field of nanotechnology is rapidly emerging as it has elicited much interest among the research community due to a diverse range of applications in different fields such as industry, medicine, and cosmetics. Rather than the bulk materials their nanostructured counterparts display unique optical, physiochemical, electrical and magnetic characteristics. This is attributed to their higher surface to volume ratio, size and shape effects. This innovative feature of nanoparticles enables them to be used extensively for biomedical applications. Different types of nanoparticles including metallic (Liao, Nehl & Hafner, 2006), fluorescent (quantum dot), magnetic (Chatterjee, Gnanasammandhan & Zhang, 2010; Corr, Rakovich & Gun’ko, 2008), protein-based nanoparticles (Hawkins, Soon-Shiong & Desai, 2008; Kogan et al., 2007) and polymeric (Kumari, Yadav & Yadav, 2010; Soppimath et al., 2001) nanoparticles are utilized for biomedical applications. However, most of the research has been focused on developing the magnetic nanoparticles. The dimension of the magnetic nanoparticles can be few nanometers up to tens of nanometers. Usually, they have a similar or smaller size than the protein molecule; it is easier for the cells or viruses to interact or attach/infiltrate inside the biological matrix of concern (Varadan, Chen & Xie, 2008).

The functionalized IONP can interact and bind with different types of biological molecules including enzymes, proteins, nucleotides or antibodies. It can even interact with the drugs based on functionalization techniques. Thus, an external magnetic field can be used to release it inside the targeted tissues, organ or a tumor (Chorny et al., 2010; Gao, Gu & Xu, 2009; Gupta & Gupta, 2005; Lacramioara et al., 2016; Laurent et al., 2008). It can be used as a curing agent for cardiovascular diseases (Chorny et al., 2010). It has the potential for curing oxidative damage (Erica, Daniel & Silvana, 2011). The presence of magnetic nanoparticles can ensure targeted drug delivery to specific organs (Verma et al., 2013). The presence of reactive oxygen species (ROS) inside the body can damage several biological activities including DNA-protein cross-links, protein fragmentation/oxidation and enzyme activation/deactivation (Kareem et al., 2015). The endogenous antioxidants are naturally produced inside the human organ. Some of the anti-oxidants can be provided externally through food and termed as exogenous antioxidants (Yehye et al., 2015). Recently the development of biocompatible nanoparticles having antioxidant properties has gained a great deal of attention.

Among different types of IONP, Magnetite (Fe_3_O_4_) nanoparticles are extensively used in magnetic separation, targeted drug delivery, magnetic resonance imaging, tissue engineering, bio-separation, magnetic hyperthermia and cell tracking (Mirzajani & Ahmadi, 2015; Thomas et al., 2015; Torres et al., 2010; Tudisco et al., 2015). Earlier research *in vivo* has demonstrated that Magnetite nanoparticles are comparatively benign due to their non-accumulating tendencies inside the vital organs. It can be promptly eliminated from the body (Boyer et al., 2010). Polymeric coating such as polyethylene glycol (PEG) over the IONP can reduce its’ toxicity level when used for human fibroblasts (Wang et al., 2008). Thus, numerous process optimization techniques have been undertaken to functionalize or coat IONPs. This has been done mainly by controlling the synthesis parameters or choosing suitable groups to incorporate with them (Barreto et al., 2011).

Flavonoids are hydrophobic substances and used as natural antioxidants in several studies. This can be classified as flavones, flavonols, flavanones, flavan-3ols, anthocyanidins, and isoflavones (Ross & Kasum, 2002). Quercetin is a kind of natural flavonol and can be extracted from berries, tea, red wine apples, citrus fruits, and red onions. It has exhibited antioxidant (Casas-Grajales & Muriel, 2015; Gormaz, Quintremil & Rodrigo, 2015), anti-inflammatory, anti-obesity, (Williams et al., 2013) anticancer (Khan et al., 2016), anti-viral and antimicrobial properties (Aziz et al., 1998; Liu et al., 2017). The coplanar structure coupled with their hydrophobicity enables them to interact with phospholipid bilayer of bio-membranes. The -OH and -C_6_H_5_ groups of flavonol can be specific or non-specific in binding to the functional proteins (enzymes, hormone receptors, and transcription factors). However, quercetin is sparingly soluble in water and unstable in physiological systems (Sun et al., 2015). Thus, its direct applications are somewhat restricted. To resolve these limitations, quercetin can be used as a functionalizing agent for nanoparticles. For instance, magnetite-quercetin nanoparticles have been studied as a drug delivery system (Barreto et al., 2011). Quercetin functionalized rare earth oxides have been demonstrated to exhibit synergistic antibacterial and hydroxyl radicle scavenging properties (Wang et al., 2013). Quercetin and Gallic acid have been used for consecutive coating of the bimetallic nanoparticles. The coating enables it to be used successfully as antioxidant, antimicrobial and antitumor agents (Mittal, Kumar & Banerjee, 2014). The coating provided by quercetin can give a protective layer over the nanoparticles to inhibit cellular damage, cytotoxicity and apoptotic death (Sarkar & Sil, 2014).

In this research, we have prepared quercetin functionalized IONP, using *in-situ* synthesis and post-synthesis method. Both the methods used here provided nano-particle samples with controlled particle sizes. The functionalization has been carried out successfully and the sample has shown great potential to be used as an antimicrobial and antioxidant agent. The antioxidant activity of the synthesized sample has been checked using 2, 2-diphenyl-1-picrylhydrazyl (DPPH) assay. Some commonly available pathogens which can easily resist different types of drugs have been chosen for antibacterial studies (e.g., Gram-positive *Staphylococcus aureus*, *Bacillus subtilis,* and Gram-negative *Escherichia coli*) *in vitro*. The antifungal activity of IONP@Q against *Aspergillus niger, Candida albicans, Trichoderma* sp. and *Saccharomyces cerevisiae* has been investigated. The biological activity of the synthesized sample has been analyzed using the PASS program. The values of the half maximal inhibitory concentration (IC50) of the DPPH antioxidant assay decreased using the functionalized one and it exhibited a 2–3 fold decreasing tendency than the unfunctionalized IONP. MIC values confirm that functionalized IONP@Q have excellent antibacterial against strains used and fungal strains. Our findings illustrated that the synthesized quercetin functionalized nanoparticles can be a promising candidate as a nano antioxidant and an antimicrobial therapeutic agent.

## Experimental Method

### Chemicals

Ferrous chloride tetrahydrate (FeCl_2_.4H_2_O, Merck), ferric chloride hexahydrate (FeCl_3_.6H_2_O, Sigma ≥ 97%), Quercetin (R & M) and ammonia solution (R & M, 28%) were used to synthesize the samples. The chemicals thus obtained were of analytical grade. Thus, those were used without further purification. Deionized water was used throughout the entire study. The surface morphological features with a particle size of IONP@Q samples were studied using a JEOL JEM-2100F High-Resolution Transmission Electron Microscope with a field emission gun operating at 200 kV. Samples for TEM measurements were prepared by evaporating a drop of the colloid onto a carbon-coated copper grid. The mean particle size of the was calculated as from the HRTEM image from an observation of 100 particles using Gatan Digital MicroGraph software.

The identity of the phase and the degree of crystallinity of the magnetite samples were investigated using a PANalytical X-ray diffractometer (model EMPYREAN) with a primary monochromatic high-intensity Cu-K*α* (*λ* = 1.54060 A°) radiation. A range of 2*θ* (from 10.00 to 90.00) was scanned. FTIR of the samples was recorded using a Perkin Elmer FTIR-Spectrum 400. EDX was studied using EDX (INCA Energy 200 (Oxford Inst.)) under a vacuumed condition with a working distance of 6 mm. The surface area method was employed to calculate the percentage composition. Raman spectra of the synthesized samples were analyzed using 514 nm Argon gas laser. The magnetism hysteresis loop measurement was conducted using Lake Shore vibrational sample magnetometer (VSM) in the solid state. The measurement was carried out under room temperature where the magnetic field range was kept at −10 to +10 kOe.

### Preparation of IONP

Both Ferrous and Ferric salts having a molar ratio of 1:1.5 were first dissolved in 100 ml deionized water (DI). NH_4_OH (3.0 M) was added dropwise (5 mL min^−1^) to the ferrous/ferric solution. The mixture was stirred at 600 rpm and the final pH of the solution was kept at 11 for 90 min, the solution thus obtained was stirred and heated to 80 °C under oxidizing environment. At the final stage of the reaction, a black precipitate was formed, and it was isolated by magnetic decantation. The precipitate was consecutively washed with deionized water (DI) and ethanol. The resultant sample thus obtained was freeze-dried.

### Functionalization using *in-situ* technique

#### Preparation of organic IONP@Q1

Ferrous and ferric salts with a molar ratio of 1:1.5 were dissolved in 100 ml DI. Quercetin dihydrate (1 g) was dissolved in 3 ml of acetone. Subsequently, the quercetin solution was added to the ferrous/ferric solution with continues stirring. NH_4_OH (3.0 M) was added dropwise (5 mL min^−1^) to the ferrous/ferric solution at 600 rpm until the solution has a final pH value of 11. The reaction was carried out at 80 °C with continuous stirring for 90 min under the oxidizing environment. The resultant magnetite nanoparticles were washed thrice with DI water and acetone collected using an external magnet and dried using freeze drier.

### Post functionalization

#### Preparation of IONP@Q2

Quercetin functionalized IONP were synthesized by the nanoprecipitation method (Kumar et al., 2014) with some modifications. Quercetin (0.5 g) was dissolved in minimum amount of acetone. Then, 1 g of IONP were dissolved in 50 ml of DI and sonicated for 30 min. Quercetin solution was continuously added during sonication followed by stirring for 24 h. The resultant magnetite nanoparticles were washed thrice with DI water and acetone collected using an external magnet and dried using freeze drier.

### Antioxidant activity

A standard DPPH method with some minor modification was carried out to observe the antioxidant activity of the synthesized sample (Deligiannakis, Sotiriou & Pratsinis, 2012; Sotiriou, Blattmann & Deligiannakis, 2016). 300 µL of sample stock methanolic suspensions and 1 mL of a methanolic solution of DPPH (0.2 mM) were mixed. The mixture was placed inside the 1 cm quartz cuvettes. After 30 min, absorbance was recorded. The absorbance was decreasing at 517 nm continuously. The experiments were performed in duplicate. The sample and the DPPH solution were allowed to mix exactly for 30 min. The absorbance measurements were then taken out precisely within 30 min after mixing. The radical scavenging activity is expressed in percentage by the given relation:

\begin{eqnarray*}\mathrm{Percentage~ of~ Inhibition} (\text{%})= \frac{(Ac-As)}{Ac} \times 100. \end{eqnarray*}

In which As = Absorbance of the compounds/ positive control and Ac = Absorbance of control (DPPH solution). To determine the concentration required to achieve 50% inhibition (IC50) of DPPH radical; the percentage of DPPH inhibition for each compound was plotted against different concentrations.

### Antimicrobial activity

#### Antibacterial activity

Agar well diffusion method was used to study the antibacterial properties of the IONP@Q. Precultures of all bacterial strains were spread on nutrient agar (NA) and wells (diameter = 6 mm) were filled with 100 µl of the test samples (100 mg/ml) and incubated at 37 °C for 24 h. Sterile distilled water was used as negative control. Positive controls used were streptomycin 100 mg/disc and ampicillin 100 mg/mL for *Gram-positive* and *Gram-negative* bacteria, respectively. Antibacterial activity i.e., the formation of halo (inhibition) zone and the diameter of inhibition zones were measured.

#### Antifungal properties

For antifungal activities of IONPQ well diffusion method was used to test it against various fungal strains. *Aspergillus niger & Trichoderma* sp, a filamentous fungus (multicellular); *Saccharomyces cerevisiae* and *Candida albican*, a yeast (unicellular);, yeast were used to study antifungal properties. Potato dextrose agar (PDA) plates were inoculated with fungal strains under aseptic conditions and wells (diameter = 6 mm) were filled with 100 µL of the test samples (100 mg/mL) and incubated at 25 °C for 48 h. Sterile distilled water was used as negative control. The positive control used was nystatin at 100 mg/mL. The percentage of inhibition (POI) of mycelia growth was calculated using Eq. (2):

\begin{eqnarray*}\mathrm{POI}= \frac{R1-R2}{R1} \times 100 \end{eqnarray*}

where

*R*1 = radius of the pathogen away from the antagonist.

*R*2 = radius of the pathogen towards the antagonist.

## Results

### Surface functional groups analysis using FTIR techniques

**Figure 1:**
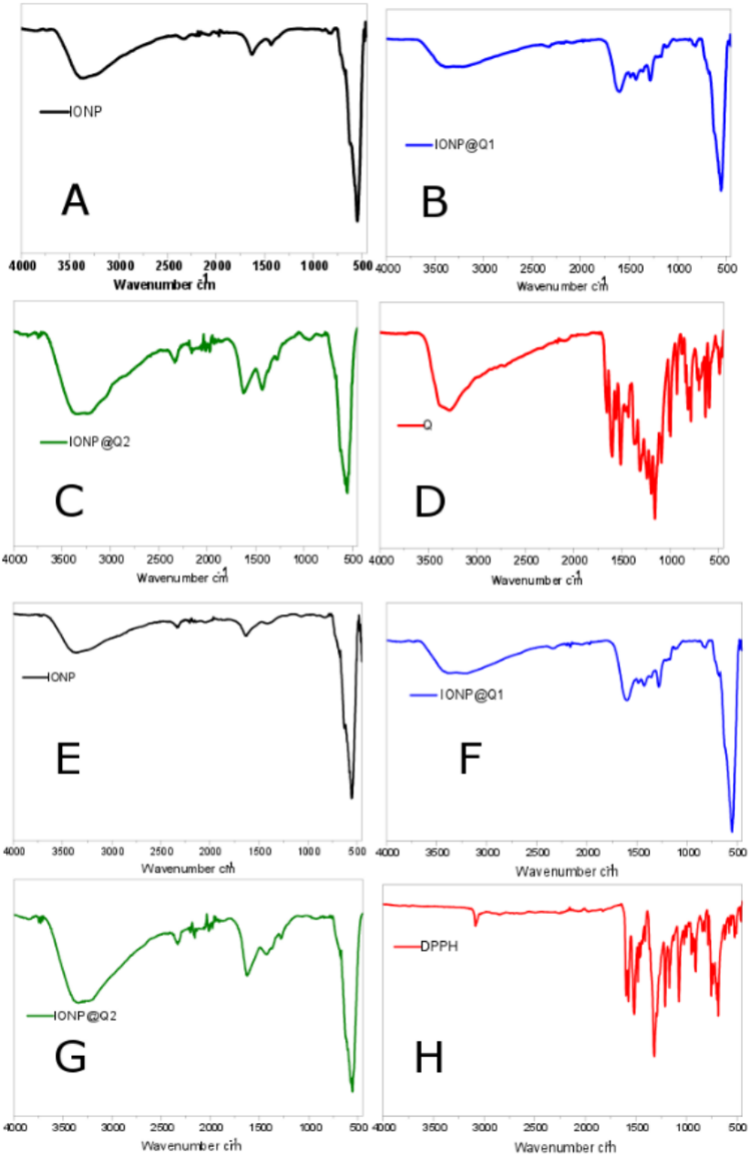
FTIR spectra of (A–D) IONP@Q before DPPH assay (E–H) IONP@Q after DPPH assay.

The surface functional groups over the synthesized samples were identified using the FTIR analysis and are illustrated in Fig. 1. Figures 1A–1D illustrates the FTIR spectra of IONPs, Q, *In-situ* IONPs and Post situ IONPs. The sharp peak around 550, 554 and 549 cm^−1^ confirms the presence of magnetite (Shah et al., 2017). After *in-situ* and post situ functionalization, the peaks were shifted slightly due to IONPs-Q complex formation. The broad peak around 3,000 cm^−1^ in all the samples showed the presence of -OH stretching vibration. The presences of the sharp peak for carbonyl groups were observed at 1,623 and 1,621 cm^−1^ IONP@Q1, and IONP@Q2, respectively (Bukhari et al., 2009). After DPPH assay was carried out, surface functional groups for IONP@Q samples were again identified and shown by Figs. 1E–1H. Excess DPPH solution was added with IONP@Q samples. The resultant mixture was stored inside the dark for 30 min. After that, the samples were washed three times with ethanol. N-O stretching band was present, and it gave rise to peaks at 1,530, 1,310, 1,513, 1,335, 1,530, 1,329 cm^−1^ for IONP@Q1 and IONP@Q2 respectively. This reflected that the functionalized surface of IONPs was attached with DPPH radical. The representative peaks for DPPH radicals were absent on IONPs (Kumar et al., 2014; Shah et al., 2017).

### Raman spectra

**Figure 2:**
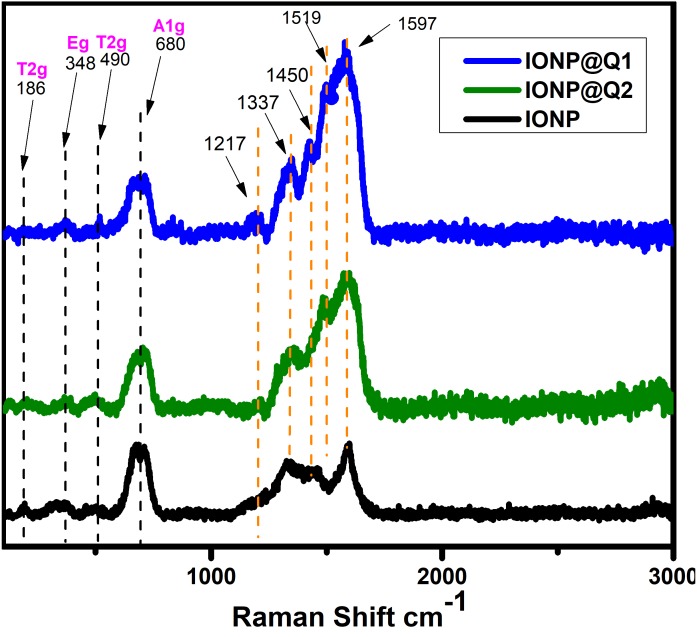
Raman spectra of IONP@Q.

The structural phase of the functionalized nanoparticles has also been illustrated by Raman spectra (Fig. 2). The Magnetite phase of IONPs is confirmed by the presence of the main vibrational mode around 671 cm^−1^ (A1g) (De Faria, Venâncio Silva & De Oliveira, 1997). Nevertheless, all the samples have the main band around 671 cm^−1^, 466 cm^−1^ and 348 cm^−1^ that have been assigned to A1g, T2g and Eg vibrations of magnetite. The absence of maghemite was also confirmed by the Raman Spectra (Francisco et al., 2011; Shebanova & Lazor, 2003). The functionalized IONPs showed some additional bands around 1,217 and 1,337 cm^−1^ which are attributed to the aromatic ring of Q. The band around 1,450 and 1,519 cm^−1^ over IONP-Q are due to the OH bending band whereas the band at 1,597 cm^−1^ indicates C=O stretching (Numata & Tanaka, 2011).

### X-ray Crystallographic Data (XRD) analysis

**Figure 3:**
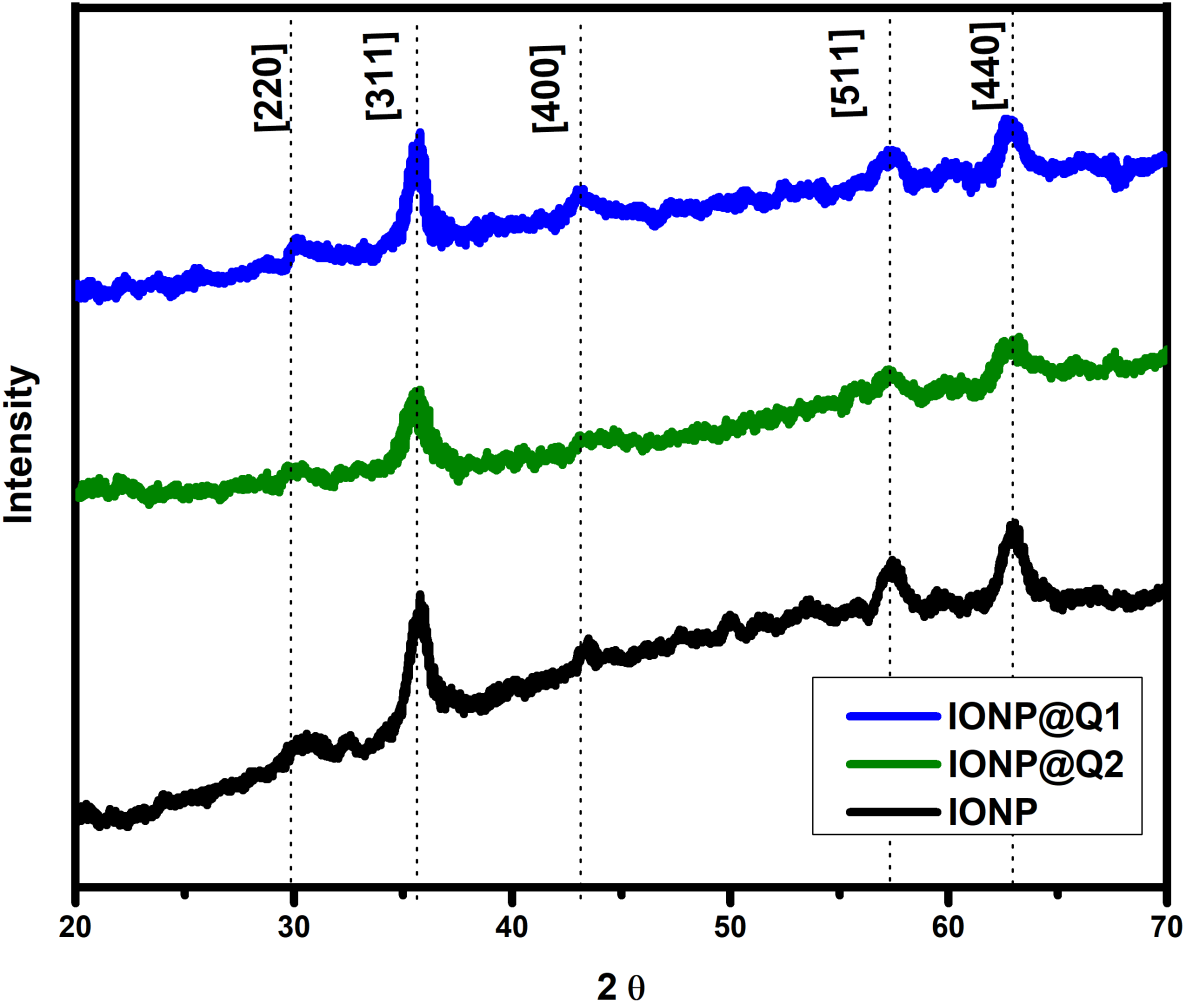
XRD spectra of IONP@Q.

The XRD curves for all the samples showed diffraction peaks at the 2 *θ* value of 30, 35, 43, 57 and 63, which is around [220], [311], [400], [511] and [440] Brag reflections, respectively (Fig. 3). The result confirmed the presence of pure magnetite nanoparticle having the cubic inverse spinal structure (JCPDS No. 82-1533) (Dorniani et al., 2012). The absence of superlattice diffractions around 210, 213, and 300 exhibits the absence of maghemite in all samples. Overall it reflected that the functionalization could not change the phase of iron oxide.

### Magnetic properties

**Figure 4:**
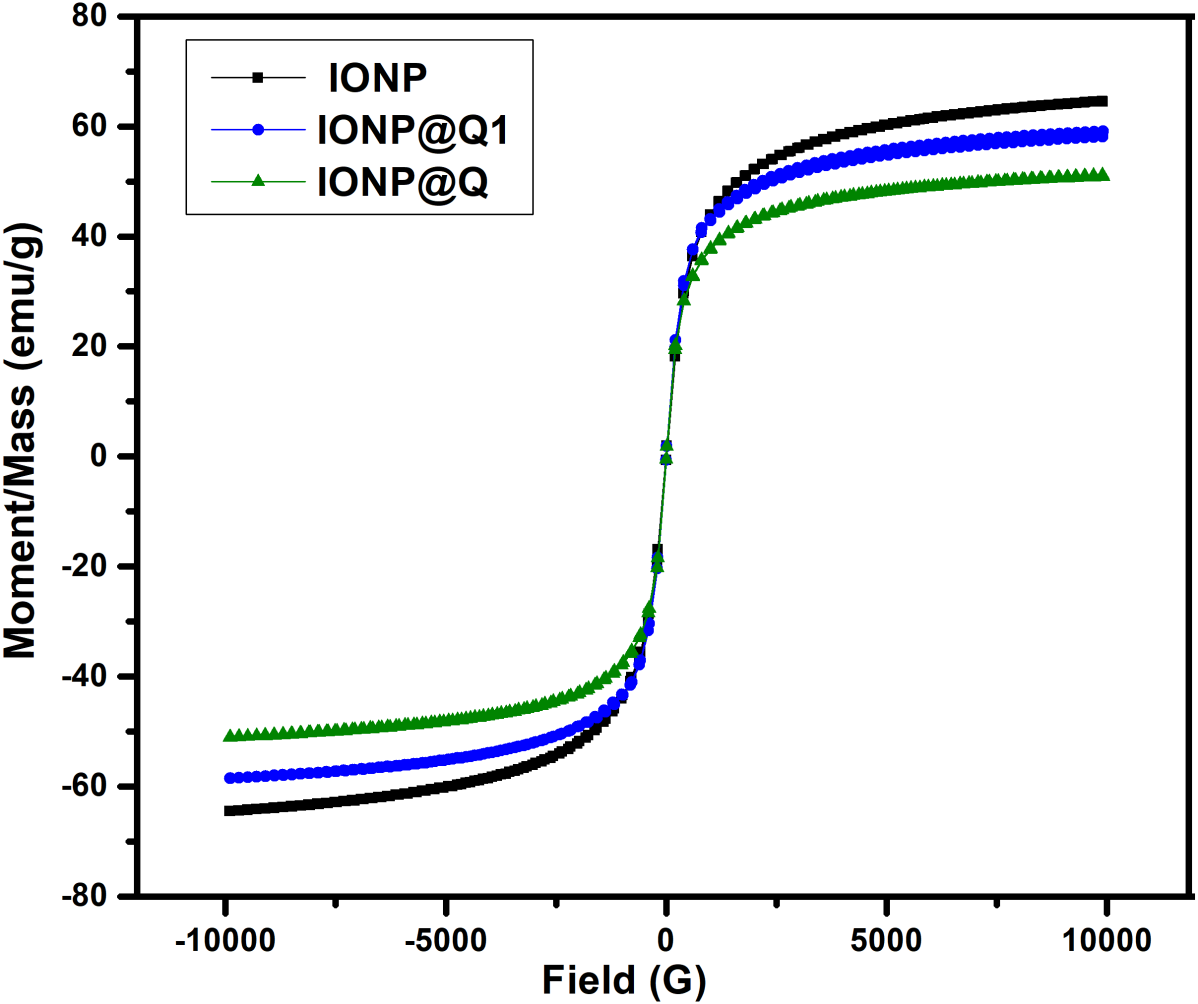
Magnetic hysteresis loops of IONP@Q.

The saturated mass magnetization was determined by vibrating sample magnetometer. The magnetization values obtained were 64.19, 51.04 and 59.07 emu g ^−1^ for IONP, IONP@Q1, and IONP@Q2, respectively. Figure 4 illustrates the hysteresis loops of the synthesized samples under the magnetic field at room temperature. Table 1 summarizes the values for the saturation magnetizations for the synthesized samples. The results reveal that the synthesized nanoparticles exhibit super-paramagnetic behavior. The functionalized samples exhibited lower saturation magnetization values than the bulk magnetite (∼92 emu g^−1^) and unfunctionalized magnetite nanoparticles (Cornell & Schwertmann, 2006). Lower magnetization values for IONP@Q is because of organic coating and impurities accumulated over the surface of the synthesized nano-composites (Dorniani et al., 2014; Dorniani et al., 2012; Ma et al., 2007).

### Morphology and structure

**Figure 5:**
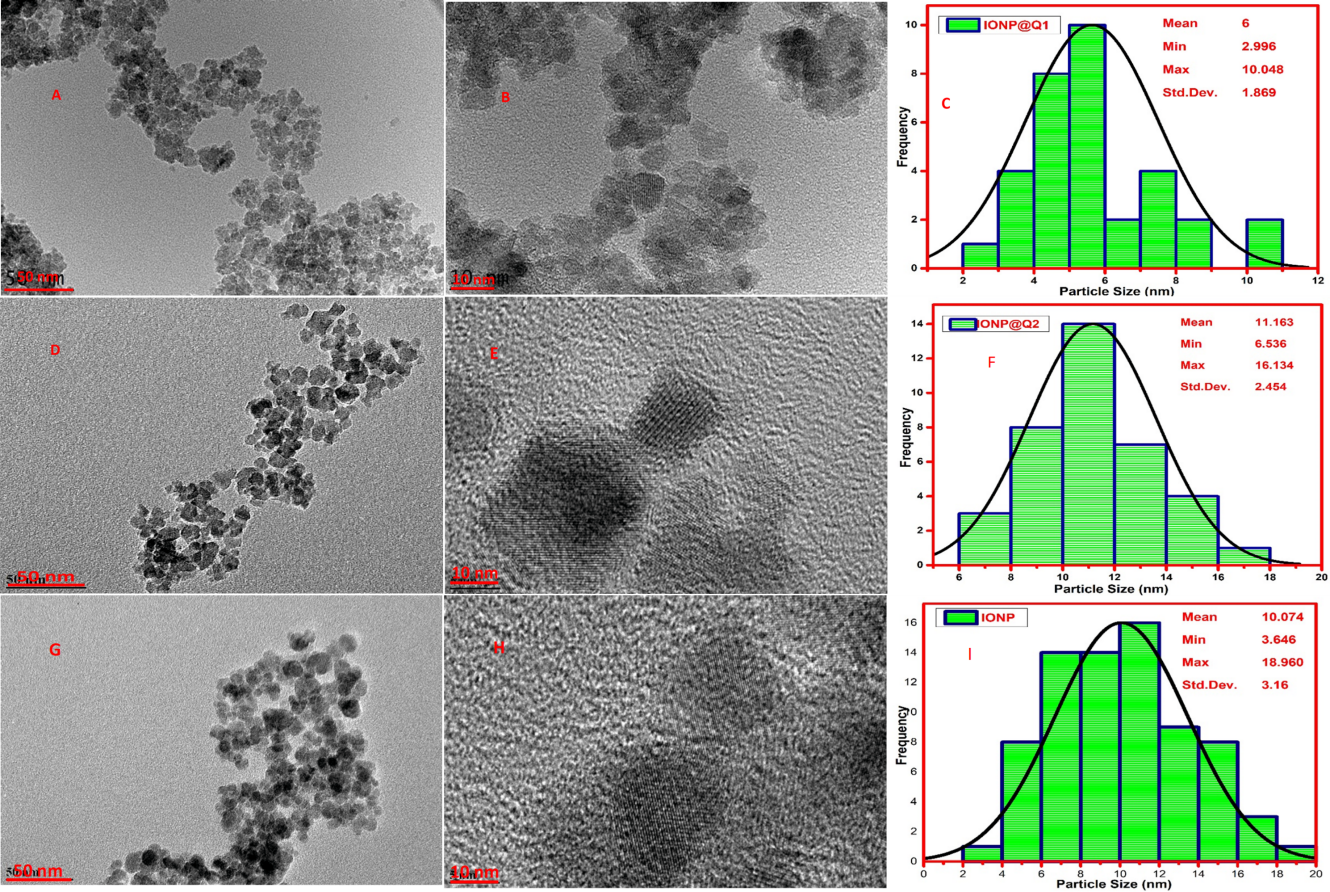
HRTEM images (A, B, D, E, G and H) and particle size distribution (C, F and I) of IONP@Q.

High-Resolution Transmission Electron Microscopy (HRTEM) was carried out to observe the particle size and morphology of the synthesized IONP@Q samples and is illustrated in Fig. 5. The average particle size observed was 10, 6 and 11 nm, for IONP, IONP@Q1, and IONP@Q2 correspondingly (Fig. 5). The particles were the spherical shape and they have uniform size distribution. The nanoparticles were aggregated to form a cluster. This occurred due to the magnetic characteristics of the synthesized particles. The crystal lattice fringe spacing 0.26 nm confirmed cubic magnetite nanoparticles (Iyengar et al., 2014).

**Figure 6:**
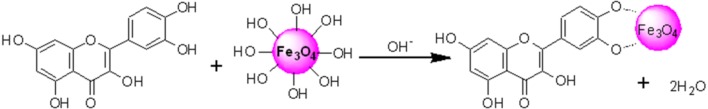
Proposed structured of the Magnetite–quercetin complex.

HRTEM images reflected that *in-situ* functionalized IONP@Q1 has a smaller particle size than the unfunctionalized IONP as well as post functionalized IONP@Q2. This reflects that the *in-situ* functionalization was efficient enough to control the size of the nanoparticles compared to other synthesis routes. This might be attributed to the formation of the metal-Q complex (Barreto et al., 2011) as shown in Fig. 6.

The presence of hydroxyl groups, over the surface of IONPs allows the attachment of different functional groups. In aqueous medium, IONPs are hydroxyl functionalized which are amphoteric and may react with acids or bases (Laurent et al., 2008).

### EDX analysis

**Figure 7:**
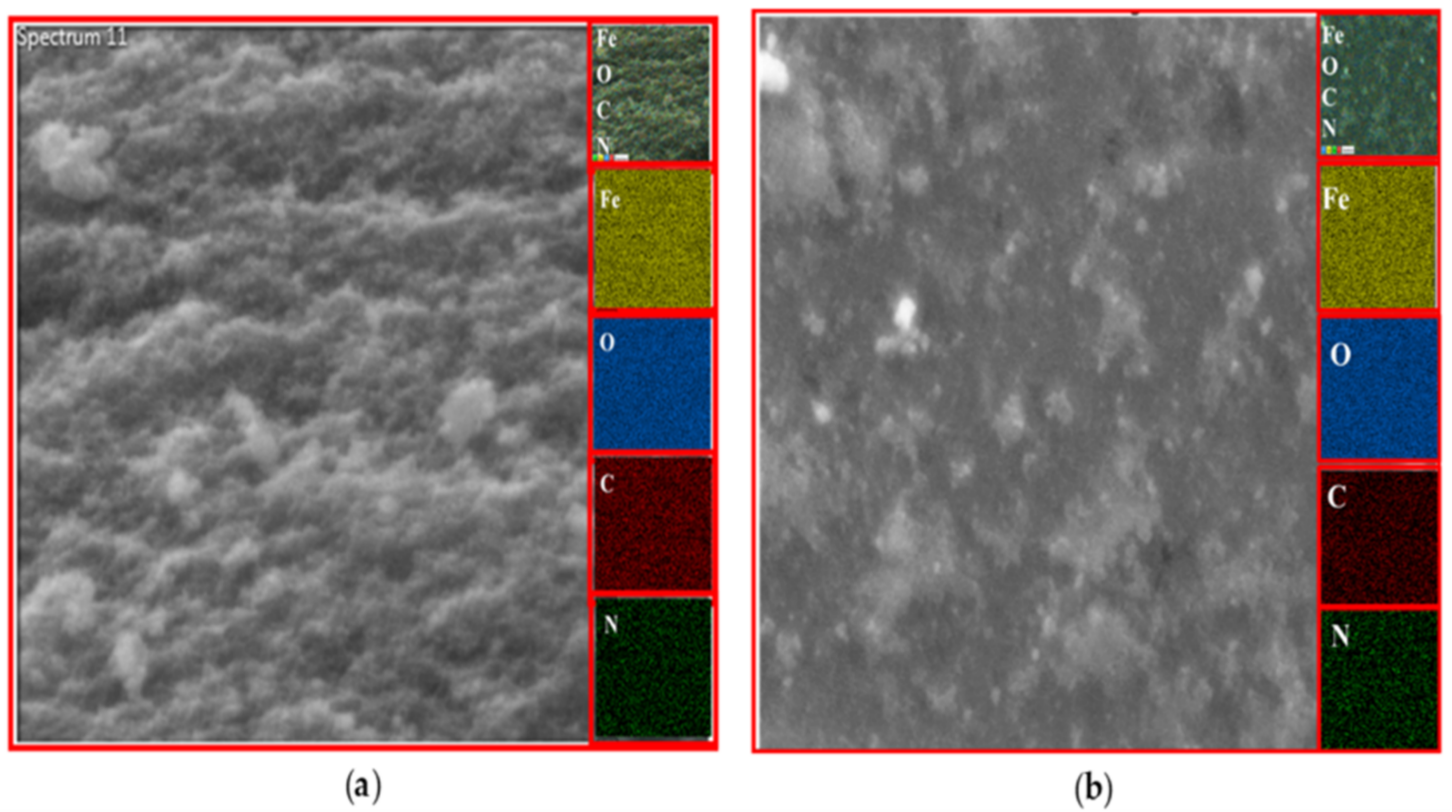
FESEM image (inset: EDX elemental map of Fe, O, C and N) of IONP@Q1 for the following elements: Fe, O, C and N (A) before DPPH assay and (B) after DPPH assay.

**Figure 8:**
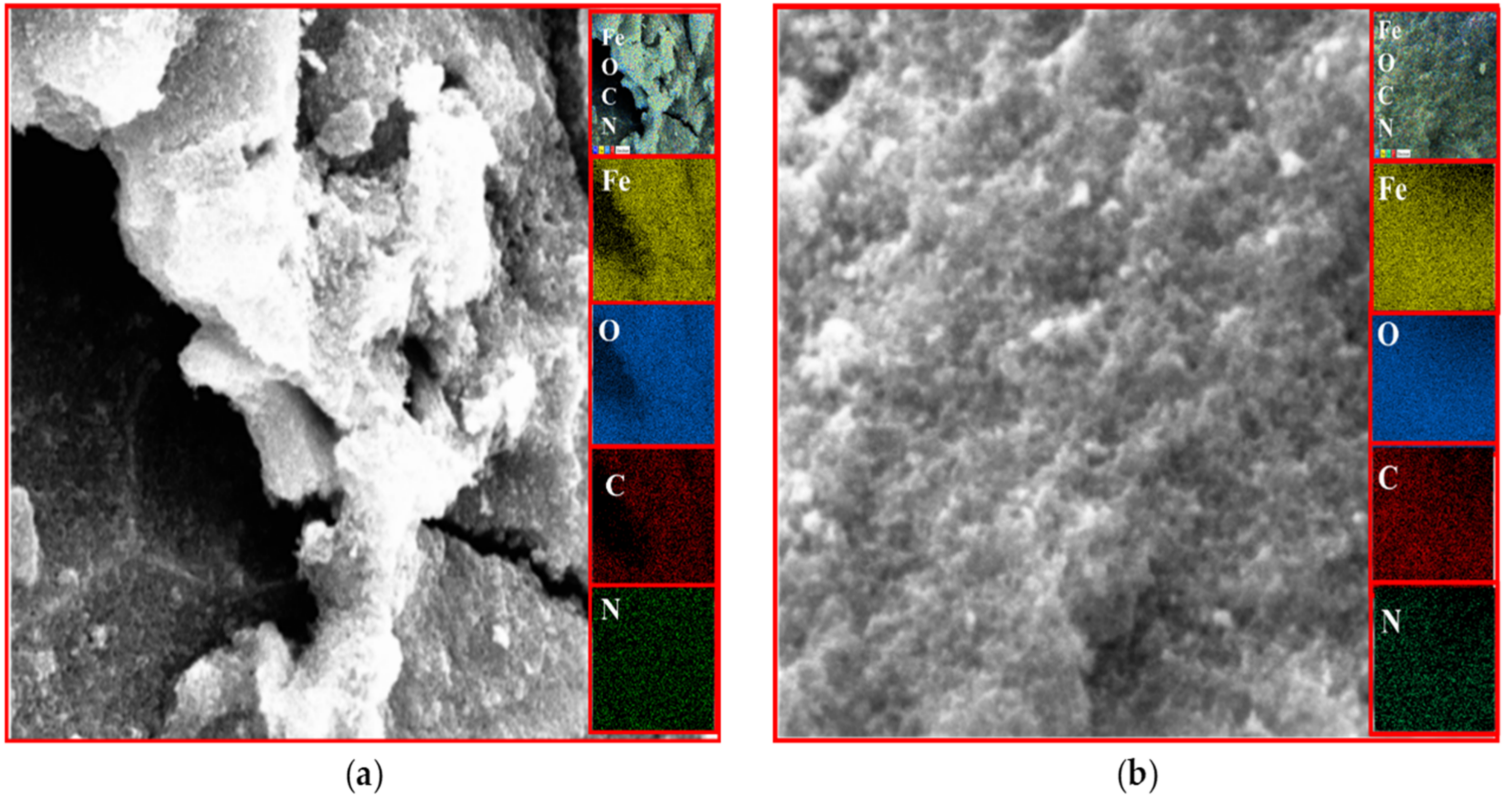
FESEM image (inset: EDX elemental map of Fe, O, C and N) of IONP@Q2 for the Fe, O, C and N (A) before DPPH assay and (B) after DPPH assay.

**Figure 9:**
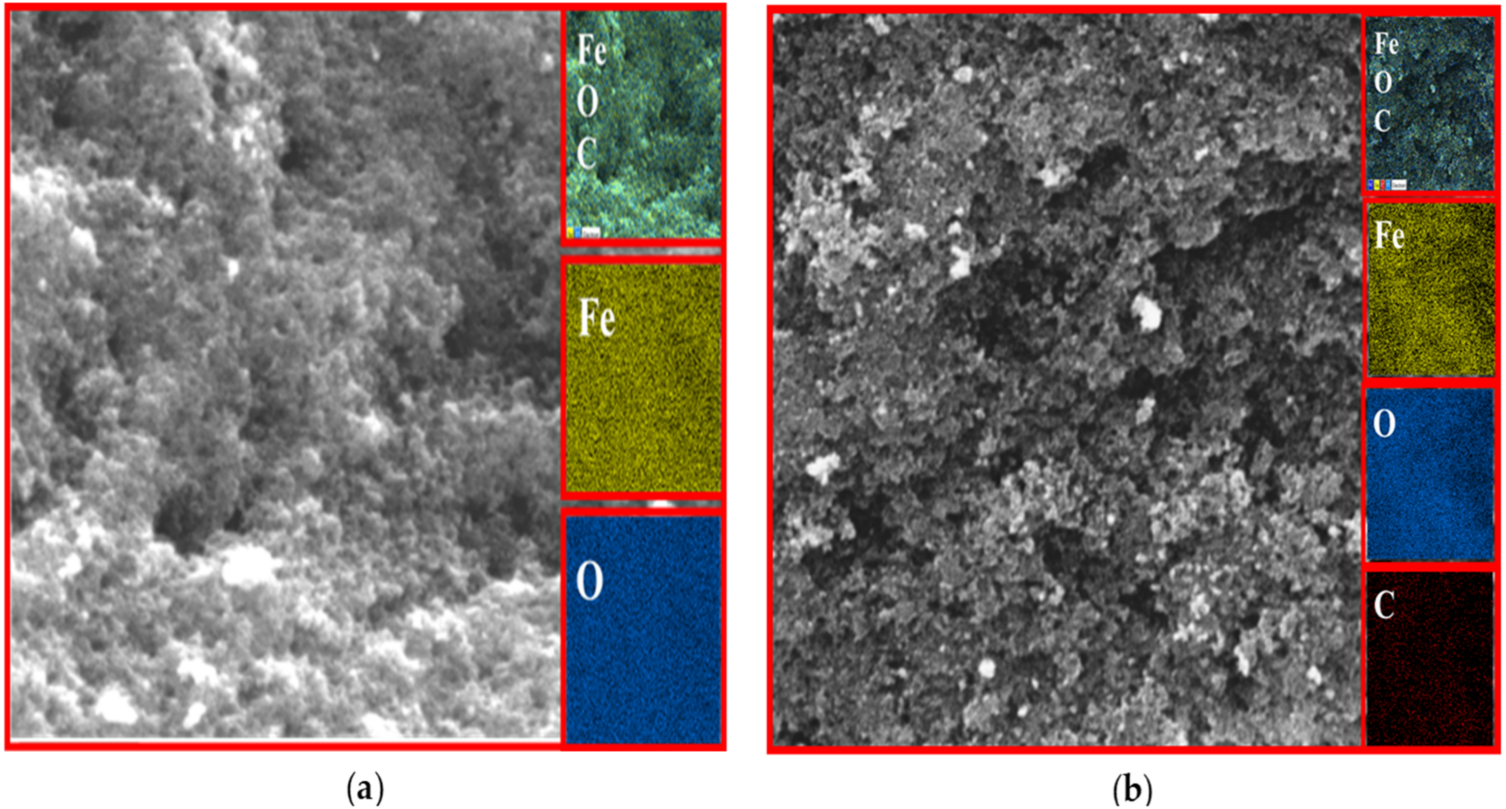
FESEM image (inset: EDX elemental map of Fe, O, C and N) of IONP for the Fe, O, C and N (A) before DPPH assay and (B) after DPPH assay.

The existence of magnetite was confirmed by the presence of Fe and O content inside the sample. The organic phase is confirmed by the presence of C contents as shown in Table 2. The uniform distribution of the atoms was identified using the mapping images (Figs. 7, 8 and 9A). After DPPH assay, N signals were also observed due to the attachment of DPPH which agrees with the FTIR. Figures 7B, 8B and 9B show the EDX of IONP@Q1, IONP@Q2, and IONP respectively. Percentage of Nitrogen increased from 0 to 0.3 and 0 to 0.5 for IONP@Q1 and IONP@Q2 respectively, while carbon contents increased from 4.6 to 7.6, and 6.1 to 8.3 f for IONP@Q1 and IONP@Q2 respectively. However, nitrogen content was not varied for unfunctionalized IONP. This reflected that the free radicals failed to attach with the IONP surface.

**Table 1:** Magnetic properties.

Sample	Ms
IONP	64.19
IONP@Q1	51.04
IONP@Q2	59.07

### PASS-predication

For designing novel free radical inhibitors for antioxidant drugs PASS prediction is useful (Kareem et al., 2015). PASS analysis can predict more than 4,000 kinds of biological activity having a mean accuracy of about 95% (Anzali et al., 2001; Kadir et al., 2014; Kadir et al., 2013; Stepanchikova et al., 2003). The results are depicted as the values obtained for appropriate Pa (probability “to be active”) and Pi (probability “to be inactive”) ratio. It is rational that the compounds having values Pa > Pi can exhibit those types of activities (Way2drug, 2015). Table 3 summarizes some portion of the predicted biological activity spectra (Lipid peroxidase inhibitor, antioxidant, a free radical scavenger, and anti-inflammatory) for the Q functionalized samples. Probable activities by PASS to be validated by experimental bioassay. Only predicted antioxidant and free radical scavenging activities of the PASS program were experimentally verified by DPPH assay. Pa > 0.7 indicated that the corresponding compound was very likely to reveal activity in experiments, 0.5 < Pa < 0.7 suggested that the compound was likely to reveal activity in experiments, while Pa < 0.5 implied that the compound was unlikely to reveal activity in experiments. However, predictive antioxidant values and other biological activities for the Q with Pa > 0.7, indicated that Q functionalized IONP surface could exhibit improved activity than the unfunctionalized iron oxide. This is due to its’ biocompatibility which can aid in drug transportation system as well as bioimaging. Activities predicted by PASS were validated by experimental bioassay.

**Table 2:** EDX elemental composition (A) Before DPPH assay (B) After DPPH assay.

Sample	A				B			
	Fe	O	C	N	Fe	O	C	N
IONP	69.6	30.4	–	–	77.5	21.2	1.3	
IONP@Q1	69.7	25.7	4.6	–	63.2	28.9	7.6	0.3
IONP@Q2	69.4	24.5	6.1	–	65.2	26.1	8.3	0.5

### Antioxidant activity

**Figure 10:**
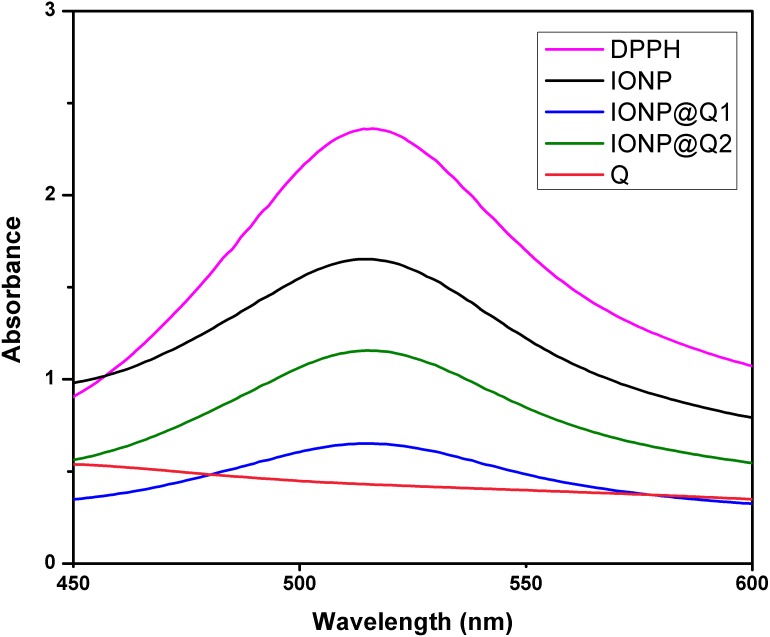
DPPH Scavenging percentage by nanomagnetite at different concentrations.

**Figure 11:**
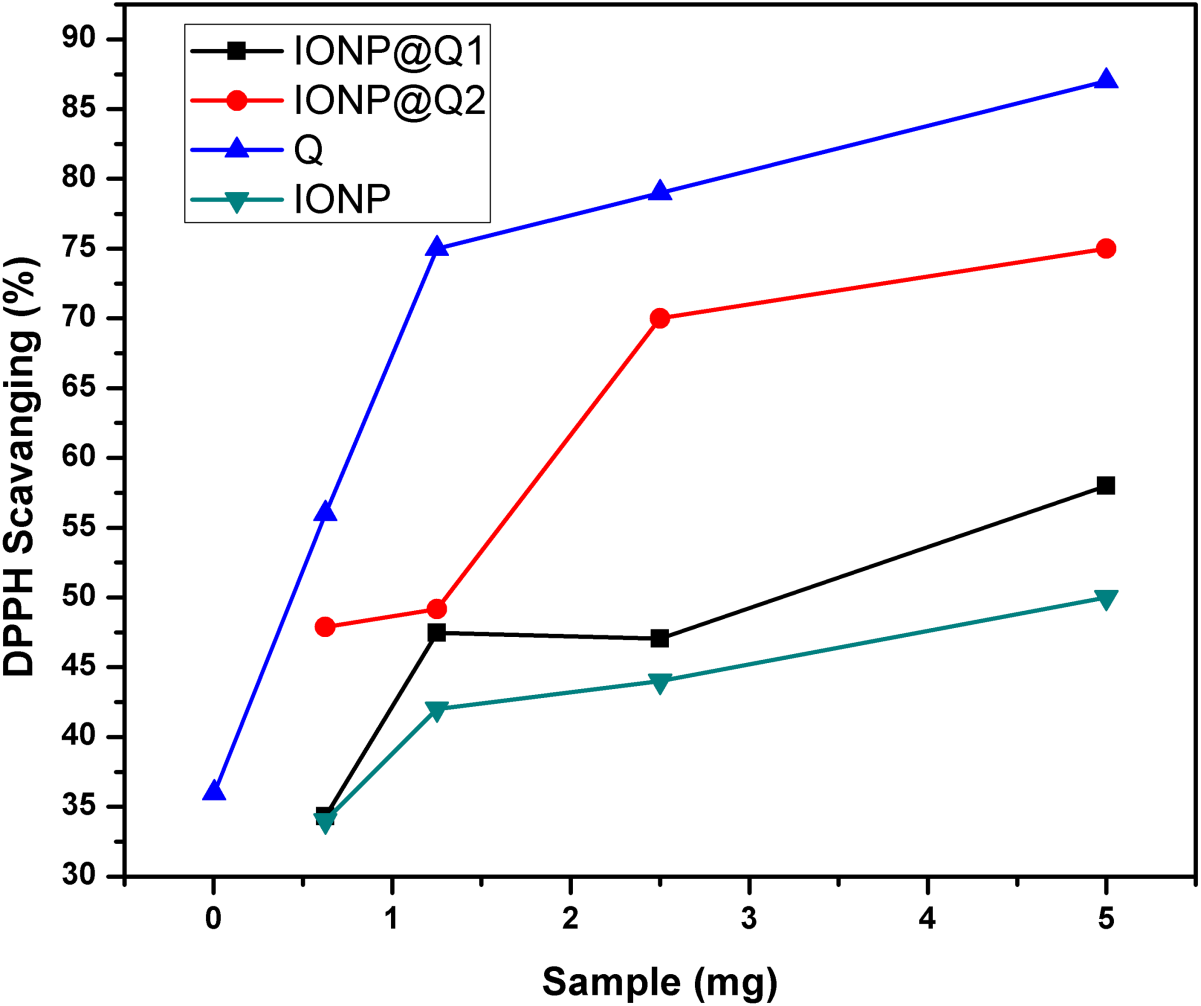
DPPH Scavenging percentage by nanomagnetite at different concentrations.

The UV–Vis absorption curve obtained for the synthesized samples are illustrated in Fig. 10. From the curve, it can be seen that the peak intensity of DPPH is continuously dropping. The decrease in absorbance around 517 nm was used to calculate the free radical scavenging percentage. Table 4 refers to the IC50 values for DPPH scavenging assay. The inhibition of stable DPPH free radicals of the *in-situ* synthesized organic nano-compounds were found to be IONP@Q1 (IC50 3 ± 0.002 mg/ml; 58%), IONP@Q2 (0.7 ± 0.002 mg/ml; 75%) and at a 10^−4^ M. The values obtained are 1–3 times more than the unfunctionalized magnetite (IC50 4.7 ± 0.002 mg/ml; 50%) (Fig. 11).

**Table 3:** Part of the Predicted Biological Activity Spectra of the Q on the Basis of PASS Prediction Software.

Biological Activity	^a^Pa	^b^Pi
Antioxidant	0.878	0.003
Free Radical Scavenger	0.816	0.002
Lipid Peroxidase Inhibitor	0.813	0.003
Anti-inflammatory	0.704	0.015

**Notes.**

a Probability “to be active”.

b Probability “to be inactive”.

**Table 4:** IC50 of IONP@Q.

IC50^a^ Values (mg) ± S.E.M^b^ and Max. inhibition %			
Sample		IC50 mg/ml	% Inhibition
IONP	5 mg	4.7 ± 0.002	50
IONP@Q1	5 mg	3.0 ± 0.002	58
IONP@Q2	5 mg	0.7 ± 0.002	75

**Notes.**

a IC50, 50% effective concentration.

b S.E.M, standard error of the mean.

The sequence followed for DPPH scavenging properties can be shown as Q > IONP@Q2 > IONP@Q1 > IONP. The free radical scavenging phenomenon can be ascribed for the transfer of electrons from IONP@Q towards the free radicals located at the central nitrogen atom of the DPPH molecule. The scavenging properties of functionalized magnetite were increased as compared with that in the case of naked IONP. Quercetin itself was more potent scavenger than both, functionalized magnetite and naked IONP. The observed moderate AOX enhancement of functionalized magnetite could be attributed to the Q on nanoparticle surface.

### Antimicrobial activity

#### Antibacterial activity

**Figure 12:**
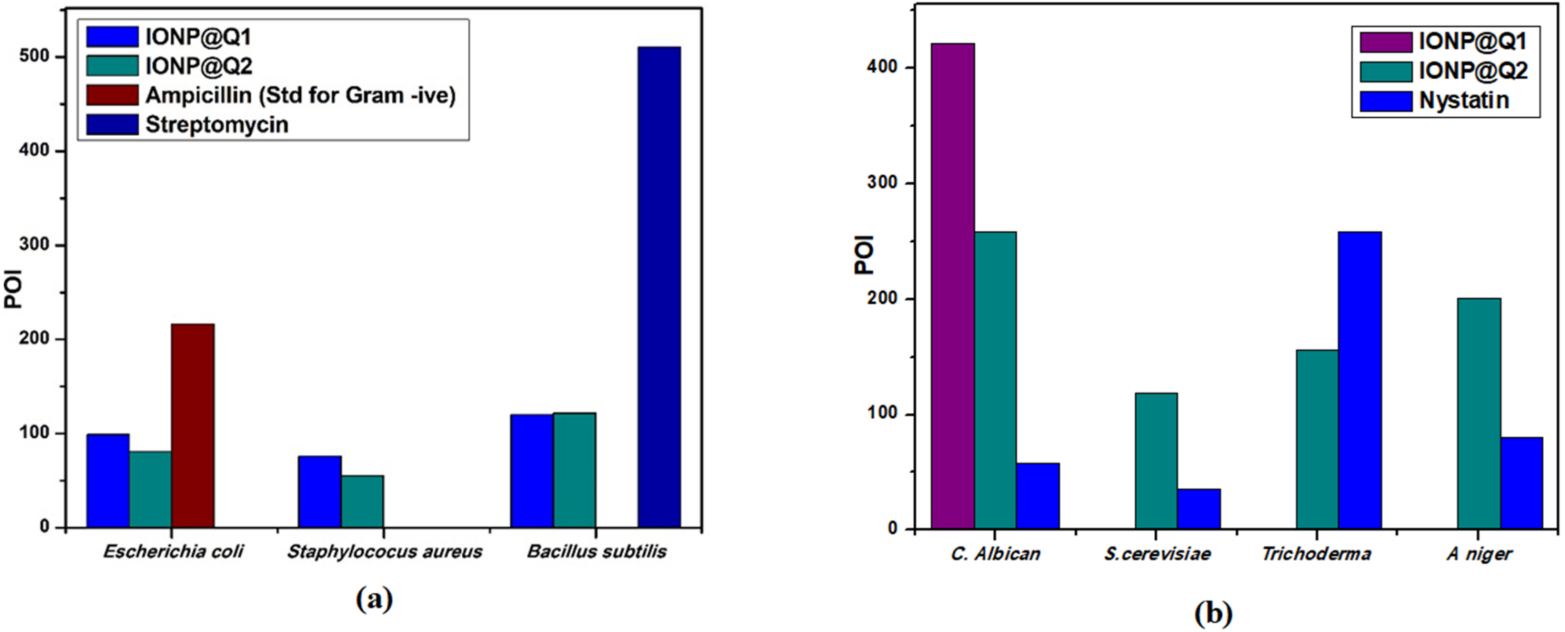
Percentage of inhibition (POI) of (A) bacterial growth and (B) fungil growth, after treatment with IONP@Q.

Agar well diffusion test was carried out and the results obtained are illustrated by Fig. 12A. The results are displayed in terms of percentage inhibition of diameter growth (PIDG) of bacterial strain using the concentration of IONP around 100 mg/ml. Both the synthesized sample functionalized using different routes exhibited antibacterial activity for both Gram-positive and negative strains. Nevertheless, the highest PIDG values were obtained after using IONP@Q2. Minimum Inhibition Concentration (MIC) is given in Table 5. This showed its’ efficient and prevailing bactericidal activity. Gram-positive bacteria have a thick peptidoglycan layer (10–30 nm), whereas Gram-negative bacteria have an additional outer layer with a thin peptidoglycan layer (10 nm). IONP@Q has shown different antibacterial activity for different bacterial strains. This was expected and can be explained depending on their composition of the cell wall for each type of strain. After adding the IONP@Q nanoparticles, bacterial growth inhibition will take place. This phenomenon takes place due to the internalization of functionalized IONPs inside the cell. This would destroy the cell wall by damaging the 1, 4 glycosidic bonds.

#### Antifungal activity

Agar well diffusion test was carried out and the results obtained are illustrated by Fig. 12B. The antifungal activity of IONP@Q against *Aspergillus niger*, *Candida albicans*, *Trichoderma* sp. and *Saccharomyces cerevisiae* was analyzed. The potential antifungal activity of the synthesized compounds among all fungi strains used. IONP@Q2 compound had exhibited the highest percentage of inhibition (POI) for *Aspergillus niger*, *Candida albicans,* and *Saccharomyces cerevisiae*, while IONP@Q1 showed highest fungal activity against *Trichoderma* sp. MIC values reveal that antifungal activity of activity of IONP@Q2 was higher for some fungal strains compared to IONP@Q1 as shown in Table 6. The nanoparticles were attached with the respiratory system which leads to the antifungal activity of the synthesized sample. The attachment caused cell death (Rudramurthy et al., 2016). In general, smaller nanoparticles exhibit higher antimicrobial activity (Padmavathy & Vijayaraghavan, 2008). However, activities also depend on the formulation process and physical characteristics of nanoparticles (Slavin et al., 2017).

**Table 5:** MIC value for antibacterial strains. Both ampicillin and streptomycine were used as standard for Gram positive and Gram negative bacteria respectively.

Bacterial species	Mean value true MIC (mg/mL)		**Q**
	**IONP@Q1**	**IONP@Q2**	
*Staphylococcus aureus*	25	10	5
*Bacillus subtilis*	25	10	5
*Escherichia coli*	10	10	5

**Table 6:** MIC value for antifungal strains. Nystatin was used as standard drug.

Fungal species	Mean value true MIC (mg/mL)		**Q**
	**IONP@Q1**	**IONP@Q2**	
*Aspergillus niger*	25	25	5
*Candida albicans*	25	10	5
*Trichoderma sp.*	25	25	5
*Saccharomyces cerevisiae*	25	10	5

## Conclusions

In this research, IONP nanoparticles were successfully functionalized. Both *in-situ* and post-situ technique was used for functionalization. HRTEM analysis was carried out to determine the average particle size for both the sample functionalized in different techniques. For *in-situ* functionalized IONP, the particle size obtained was 6 nm whereas, after post-functionalization, the synthesized IONP@Q2 had the particle size 11 nm-slightly bigger than the *in-situ* one. The particle size obtained here followed the sequence of IONP@Q2 > IONP > IONP@Q1. Due to the use of the *in-situ* process, the particle size of IONP was controlled by Q. This is owing to the formation of a kind of IONP-Q complex. Predictive values for antioxidant activities and other biological activities for the Q with Pa > 0.7 were presented. Owing to its biocompatibility, it can be used as a promising candidate for drug delivery and bio-imaging agent. In this research, probable activities by PASS were validated by experimental bioassay. The scavenging activity of the sample using the DPPH was found to be in the order of IONP@Q2 > IONP@Q1 > IONP. Both the methods used here for functionalization of IONP has increased its’ free radical scavenging capacity more than one- to three- fold compared to the unfunctionalized one. The synergistic effect of the magnetite coated by Q had not only increased the free radical scavenging capacity but also controlled the particle size of IONP. The formation of IONP@Q-DPPH was confirmed by FTIR and EDX. Finally, IONP@Q showed potential antifungal and antibacterial effects on the strains under observation. The antimicrobial of IONP@Q2 was higher than IONP@Q1. This might be taken place through the destruction of the membrane. The findings here clearly reveal that the synthesized IONP@Q has combinatorial properties (magnetic, antioxidant and antimicrobial). Thus, the application of the synthesized sample can be promising for further clinical applications.

##  Supplemental Information

10.7717/peerj.7651/supp-1 Data S1 EDX Spectra and Pass Studies of IONP@Q

## Additional Information and Declarations

### Data Availability

The following information was supplied regarding data availability: The raw data is available as Supplemental File.
