# Supplementary material for: Magnetically directed antioxidant and antimicrobial agent: synthesis and surface functionalization of magnetite with quercetin
Source: PeerJ. 2019 Nov 20;7:e7651. doi: 10.7717/peerj.7651 (PMC6874855; doi:10.7717/peerj.7651)
Supplement: Data S1 [file peerj-07-7651-s001.docx]

**Supporting Information**

**Journal:**

**Title: “****Magnetically Directed Antioxidant and Antimicrobial Agent; Size-Controlled Synthesis and Surface Functionalization of magnetite with Quercetin”**

| A | B |
| --- | --- |
| 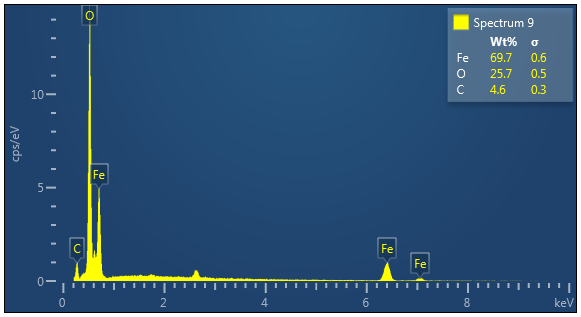 | 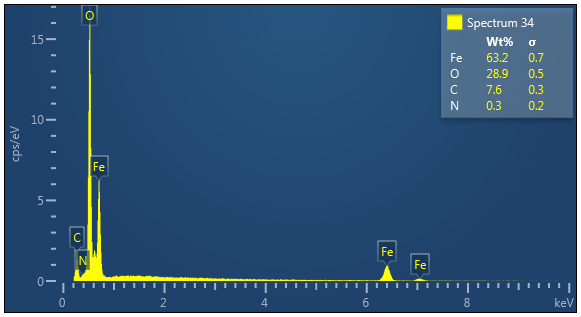 |

Figure S1 EDX spectrum of IONP@Q1 A) Before DPPH assay B) After DPPH assay

| A | B |
| --- | --- |
| 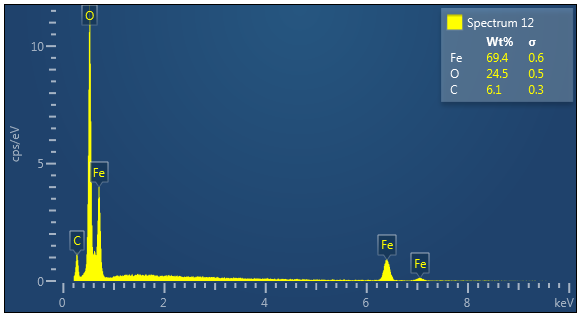 | 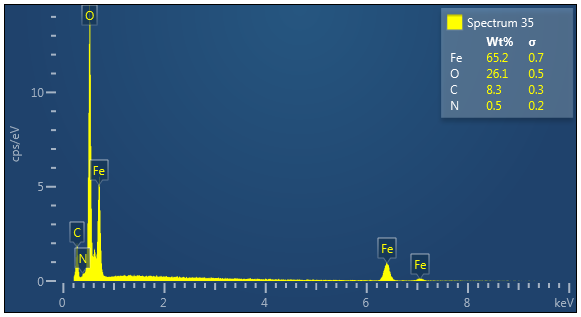 |

Figure S2 EDX spectrum of IONP@Q2 A) Before DPPH assay B) After DPPH assay

| A | B |
| --- | --- |
| 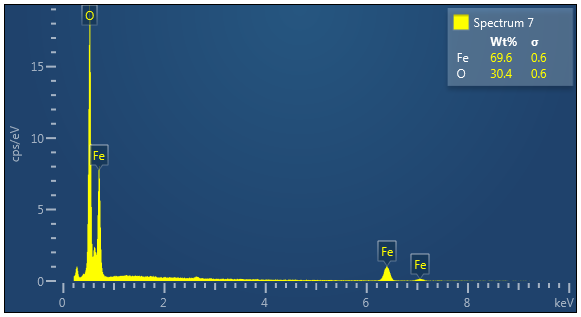 | 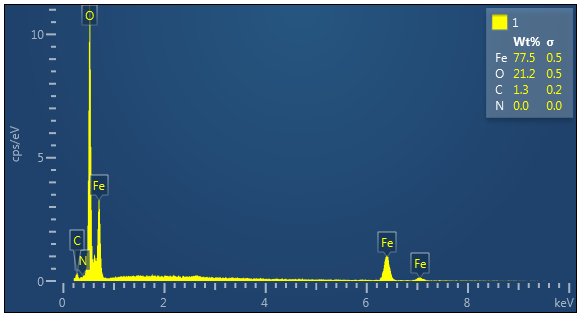 |

Figure S3 EDX spectrum of IONP A) Before DPPH assay B) After DPPH assay

Table S2. PASS result for Q

| Pa | Pi |  |
| --- | --- | --- |
| 0,955 | 0,002 | Arylacetonitrilase inhibitor |
| 0,954 | 0,002 | Chlordecone reductase inhibitor |
| 0,950 | 0,002 | Dehydro-L-gulonate decarboxylase inhibitor |
| 0,950 | 0,003 | Testosterone 17beta-dehydrogenase (NADP+) inhibitor |
| 0,944 | 0,002 | Glutathione thiolesterase inhibitor |
| 0,943 | 0,002 | Alkane 1-monooxygenase inhibitor |
| 0,941 | 0,003 | Sugar-phosphatase inhibitor |
| 0,938 | 0,002 | NADPH-cytochrome-c2 reductase inhibitor |
| 0,934 | 0,001 | Threonine aldolase inhibitor |
| 0,934 | 0,001 | Catechol 1,2-dioxygenase inhibitor |
| 0,933 | 0,002 | 2-Hydroxyquinoline 8-monooxygenase inhibitor |
| 0,933 | 0,002 | Glucan endo-1,6-beta-glucosidase inhibitor |
| 0,932 | 0,002 | Arylsulfate sulfotransferase inhibitor |
| 0,930 | 0,002 | Ribulose-phosphate 3-epimerase inhibitor |
| 0,929 | 0,002 | 2-Nitropropane dioxygenase inhibitor |
| 0,928 | 0,004 | Alkenylglycerophosphocholine hydrolase inhibitor |
| 0,928 | 0,003 | Monodehydroascorbate reductase (NADH) inhibitor |
| 0,926 | 0,002 | UDP-N-acetylglucosamine 4-epimerase inhibitor |
| 0,926 | 0,002 | Glutamyl endopeptidase II inhibitor |
| 0,924 | 0,001 | Phloroglucinol reductase inhibitor |
| 0,924 | 0,002 | Aspartate-phenylpyruvate transaminase inhibitor |
| 0,923 | 0,002 | Fatty-acyl-CoA synthase inhibitor |
| 0,923 | 0,003 | Pullulanase inhibitor |
| 0,923 | 0,005 | Methylenetetrahydrofolate reductase (NADPH) inhibitor |
| 0,921 | 0,003 | Antiseptic |
| 0,919 | 0,003 | Taurine dehydrogenase inhibitor |
| 0,919 | 0,003 | Prolyl aminopeptidase inhibitor |
| 0,915 | 0,002 | Corticosteroid side-chain-isomerase inhibitor |
| 0,915 | 0,003 | Dextranase inhibitor |
| 0,913 | 0,002 | Bisphosphoglycerate phosphatase inhibitor |
| 0,915 | 0,004 | Ubiquinol-cytochrome-c reductase inhibitor |
| 0,912 | 0,001 | Cis-1,2-dihydro-1,2-dihydroxynaphthalene dehydrogenase inhibitor |
| 0,910 | 0,003 | Arginine 2-monooxygenase inhibitor |
| 0,909 | 0,002 | Gamma-guanidinobutyraldehyde dehydrogenase inhibitor |
| 0,909 | 0,002 | 5-O-(4-coumaroyl)-D-quinate 3'-monooxygenase inhibitor |
| 0,907 | 0,002 | Sulfite reductase inhibitor |
| 0,908 | 0,003 | 2-Dehydropantoate 2-reductase inhibitor |
| 0,905 | 0,002 | NADH kinase inhibitor |
| 0,908 | 0,006 | Aspulvinone dimethylallyltransferase inhibitor |
| 0,903 | 0,001 | Procollagen-lysine 5-dioxygenase inhibitor |
| 0,905 | 0,004 | Glucose oxidase inhibitor |
| 0,901 | 0,002 | Alcohol oxidase inhibitor |
| 0,902 | 0,004 | Feruloyl esterase inhibitor |
| 0,901 | 0,003 | Phosphatidylcholine-retinol O-acyltransferase inhibitor |
| 0,901 | 0,003 | Creatininase inhibitor |
| 0,901 | 0,004 | Aldehyde oxidase inhibitor |
| 0,898 | 0,002 | Sulfite oxidase inhibitor |
| 0,898 | 0,002 | Hyponitrite reductase inhibitor |
| 0,898 | 0,003 | 3-Hydroxybenzoate 6-monooxygenase inhibitor |
| 0,897 | 0,001 | Aminobutyraldehyde dehydrogenase inhibitor |
| 0,899 | 0,005 | Sphinganine kinase inhibitor |
| 0,898 | 0,004 | Anaphylatoxin receptor antagonist |
| 0,898 | 0,004 | Superoxide dismutase inhibitor |
| 0,896 | 0,003 | Fusarinine-C ornithinesterase inhibitor |
| 0,895 | 0,002 | Aryl-alcohol dehydrogenase (NADP+) inhibitor |
| 0,896 | 0,003 | 2-Hydroxymuconate-semialdehyde hydrolase inhibitor |
| 0,895 | 0,002 | Peptide alpha-N-acetyltransferase inhibitor |
| 0,895 | 0,003 | Dimethylargininase inhibitor |
| 0,893 | 0,003 | N-benzyloxycarbonylglycine hydrolase inhibitor |
| 0,892 | 0,002 | Methylamine-glutamate N-methyltransferase inhibitor |
| 0,890 | 0,002 | Chenodeoxycholoyltaurine hydrolase inhibitor |
| 0,891 | 0,003 | Peroxidase inhibitor |
| 0,891 | 0,003 | Trans-acenaphthene-1,2-diol dehydrogenase inhibitor |
| 0,890 | 0,002 | Electron-transferring-flavoprotein dehydrogenase inhibitor |
| 0,890 | 0,002 | Glutamine-phenylpyruvate transaminase inhibitor |
| 0,889 | 0,003 | Polyamine-transporting ATPase inhibitor |
| 0,889 | 0,002 | Crotonoyl-[acyl-carrier-protein] hydratase inhibitor |
| 0,887 | 0,001 | 2,5-Dihydroxypyridine 5,6-dioxygenase inhibitor |
| 0,886 | 0,002 | Opheline kinase inhibitor |
| 0,886 | 0,002 | Taurocyamine kinase inhibitor |
| 0,887 | 0,002 | 4-Methoxybenzoate monooxygenase (O-demethylating) inhibitor |
| 0,886 | 0,002 | Gluconate 5-dehydrogenase inhibitor |
| 0,884 | 0,001 | Astringent |
| 0,885 | 0,003 | Pterin deaminase inhibitor |
| 0,885 | 0,002 | L-glutamate oxidase inhibitor |
| 0,885 | 0,003 | Nitrate reductase (cytochrome) inhibitor |
| 0,884 | 0,002 | Magnesium-protoporphyrin IX monomethyl ester (oxidative) cyclase inhibitor |
| 0,888 | 0,007 | Glutamate-5-semialdehyde dehydrogenase inhibitor |
| 0,883 | 0,003 | L-glucuronate reductase inhibitor |
| 0,881 | 0,001 | Glyoxylate oxidase inhibitor |
| 0,882 | 0,002 | N-acetylneuraminate synthase inhibitor |
| 0,882 | 0,002 | S-alkylcysteine lyase inhibitor |
| 0,881 | 0,002 | N-acylmannosamine kinase inhibitor |
| 0,881 | 0,002 | Rhamnulose-1-phosphate aldolase inhibitor |
| 0,881 | 0,002 | Cyclohexyl-isocyanide hydratase inhibitor |
| 0,882 | 0,003 | Acetylesterase inhibitor |
| 0,881 | 0,002 | 3-Hydroxybenzoate 4-monooxygenase inhibitor |
| 0,881 | 0,002 | tRNA-pseudouridine synthase I inhibitor |
| 0,880 | 0,002 | N-Acyl-D-aspartate deacylase inhibitor |
| 0,880 | 0,002 | Glutamate decarboxylase inhibitor |
| 0,880 | 0,003 | Exoribonuclease II inhibitor |
| 0,890 | 0,014 | Membrane integrity agonist |
| 0,878 | 0,002 | Ferredoxin-NAD+ reductase inhibitor |
| 0,878 | 0,002 | Naphthalene 1,2-dioxygenase inhibitor |
| 0,878 | 0,002 | NADPH-ferrihemoprotein reductase inhibitor |
| 0,882 | 0,007 | Benzoate-CoA ligase inhibitor |
| 0,877 | 0,002 | Benzoate 4-monooxygenase inhibitor |
| 0,877 | 0,003 | Allyl-alcohol dehydrogenase inhibitor |
| 0,878 | 0,003 | Glucan endo-1,3-beta-D-glucosidase inhibitor |
| 0,876 | 0,002 | Phosphatidylserine decarboxylase inhibitor |
| 0,875 | 0,003 | Poly(alpha-L-guluronate) lyase inhibitor |
| 0,876 | 0,004 | ADP-thymidine kinase inhibitor |
| 0,873 | 0,002 | Tryptophanamidase inhibitor |
| 0,873 | 0,003 | Aldehyde dehydrogenase (pyrroloquinoline-quinone) inhibitor |
| 0,872 | 0,003 | Mucinaminylserine mucinaminidase inhibitor |
| 0,871 | 0,002 | Fructan beta-fructosidase inhibitor |
| 0,871 | 0,003 | Spermidine dehydrogenase inhibitor |
| 0,869 | 0,003 | Aspartate-ammonia ligase inhibitor |
| 0,868 | 0,002 | Long-chain-aldehyde dehydrogenase inhibitor |
| 0,870 | 0,004 | N-acetylneuraminate 7-O(or 9-O)-acetyltransferase inhibitor |
| 0,867 | 0,001 | Lactaldehyde reductase inhibitor |
| 0,868 | 0,003 | Arylalkyl acylamidase inhibitor |
| 0,868 | 0,003 | Formaldehyde transketolase inhibitor |
| 0,870 | 0,005 | NADPH peroxidase inhibitor |
| 0,867 | 0,002 | N-methylhydantoinase (ATP-hydrolysing) inhibitor |
| 0,866 | 0,003 | 4-Hydroxyproline epimerase inhibitor |
| 0,866 | 0,002 | Pyruvate decarboxylase inhibitor |
| 0,865 | 0,002 | Carbon-monoxide dehydrogenase inhibitor |
| 0,865 | 0,002 | Anthranilate 3-monooxygenase (deaminating) inhibitor |
| 0,864 | 0,001 | 2,6-Dihydroxypyridine 3-monooxygenase inhibitor |
| 0,864 | 0,002 | Catechol 2,3-dioxygenase inhibitor |
| 0,863 | 0,002 | 2-Oxoaldehyde dehydrogenase (NADP+) inhibitor |
| 0,864 | 0,003 | Amine dehydrogenase inhibitor |
| 0,867 | 0,007 | Pro-opiomelanocortin converting enzyme inhibitor |
| 0,863 | 0,003 | Urethanase inhibitor |
| 0,862 | 0,002 | Histidinol-phosphatase inhibitor |
| 0,860 | 0,001 | Gentisate 1,2-dioxygenase inhibitor |
| 0,862 | 0,003 | Xylan endo-1,3-beta-xylosidase inhibitor |
| 0,860 | 0,002 | 2-Dehydropantolactone reductase (A-specific) inhibitor |
| 0,859 | 0,001 | Phenylpyruvate decarboxylase inhibitor |
| 0,859 | 0,002 | Beta-carotene 15,15'-monooxygenase inhibitor |
| 0,858 | 0,003 | 1,4-Lactonase inhibitor |
| 0,858 | 0,003 | Cholestanetriol 26-monooxygenase inhibitor |
| 0,859 | 0,003 | Mitochondrial processing peptidase inhibitor |
| 0,858 | 0,002 | Tpr proteinase (Porphyromonas gingivalis) inhibitor |
| 0,858 | 0,003 | Phenol O-methyltransferase inhibitor |
| 0,857 | 0,002 | Uroporphyrinogen-III synthase inhibitor |
| 0,857 | 0,003 | Sarcosine oxidase inhibitor |
| 0,856 | 0,002 | Glyoxylate reductase inhibitor |
| 0,854 | 0,003 | Peptide-N4-(N-acetyl-beta-glucosaminyl)asparagine amidase inhibitor |
| 0,853 | 0,003 | 4-Nitrophenol 2-monooxygenase inhibitor |
| 0,852 | 0,003 | Anthranilate-CoA ligase inhibitor |
| 0,852 | 0,003 | Poly(beta-D-mannuronate) lyase inhibitor |
| 0,849 | 0,001 | 2-Pyrocatechuate decarboxylase inhibitor |
| 0,849 | 0,002 | Mannan endo-1,4-beta-mannosidase inhibitor |
| 0,849 | 0,002 | 6-Pyruvoyltetrahydropterin synthase inhibitor |
| 0,849 | 0,002 | 4-Chlorophenylacetate 3,4-dioxygenase inhibitor |
| 0,855 | 0,009 | Antieczematic |
| 0,850 | 0,004 | Lysine 2,3-aminomutase inhibitor |
| 0,849 | 0,005 | Fragilysin inhibitor |
| 0,846 | 0,002 | Acylphosphatase inhibitor |
| 0,846 | 0,002 | 6-Carboxyhexanoate-CoA ligase inhibitor |
| 0,846 | 0,002 | Biotin-CoA ligase inhibitor |
| 0,846 | 0,002 | Homoaconitate hydratase inhibitor |
| 0,846 | 0,002 | Triacetate-lactonase inhibitor |
| 0,847 | 0,003 | Fructose 5-dehydrogenase inhibitor |
| 0,845 | 0,002 | Glycerol dehydratase inhibitor |
| 0,846 | 0,003 | Transketolase inhibitor |
| 0,846 | 0,003 | Limulus clotting factor B inhibitor |
| 0,842 | 0,003 | Prostaglandin-A1 DELTA-isomerase inhibitor |
| 0,842 | 0,002 | Glutarate-semialdehyde dehydrogenase inhibitor |
| 0,842 | 0,003 | Pectate lyase inhibitor |
| 0,842 | 0,003 | Antimutagenic |
| 0,840 | 0,002 | 2-Oxoglutarate decarboxylase inhibitor |
| 0,840 | 0,002 | D-xylulose reductase inhibitor |
| 0,839 | 0,002 | Gamma-butyrobetaine dioxygenase inhibitor |
| 0,838 | 0,002 | 3-Hydroxy-4-oxoquinoline 2,4-dioxygenase inhibitor |
| 0,838 | 0,002 | Styrene-oxide isomerase inhibitor |
| 0,838 | 0,002 | Ornithine cyclodeaminase inhibitor |
| 0,838 | 0,002 | Aminocarboxymuconate-semialdehyde decarboxylase inhibitor |
| 0,839 | 0,004 | Levanase inhibitor |
| 0,837 | 0,002 | Guanidinoacetase inhibitor |
| 0,838 | 0,003 | Carnitinamidase inhibitor |
| 0,838 | 0,003 | Hydrogen dehydrogenase inhibitor |
| 0,837 | 0,002 | 4-Hydroxyglutamate transaminase inhibitor |
| 0,836 | 0,002 | Mannitol-1-phosphatase inhibitor |
| 0,836 | 0,002 | Salicylate 1-monooxygenase inhibitor |
| 0,836 | 0,003 | Preneoplastic conditions treatment |
| 0,835 | 0,002 | 3-Carboxyethylcatechol 2,3-dioxygenase inhibitor |
| 0,835 | 0,003 | Leucolysin inhibitor |
| 0,834 | 0,002 | Vanillyl-alcohol oxidase inhibitor |
| 0,836 | 0,004 | Phospholipid-translocating ATPase inhibitor |
| 0,833 | 0,002 | Creatinine deaminase inhibitor |
| 0,833 | 0,002 | Gluconolactonase inhibitor |
| 0,834 | 0,003 | Nicotinate dehydrogenase inhibitor |
| 0,830 | 0,001 | Shikimate 5-dehydrogenase inhibitor |
| 0,831 | 0,003 | S-formylglutathione hydrolase inhibitor |
| 0,830 | 0,002 | N-carbamoyl-L-amino-acid hydrolase inhibitor |
| 0,830 | 0,002 | Camphor 1,2-monooxygenase inhibitor |
| 0,830 | 0,003 | Aryldialkylphosphatase inhibitor |
| 0,830 | 0,003 | Laccase inhibitor |
| 0,849 | 0,024 | CYP2C12 substrate |
| 0,828 | 0,003 | 2-Haloacid dehalogenase (configuration-inverting) inhibitor |
| 0,827 | 0,003 | Opine dehydrogenase inhibitor |
| 0,825 | 0,002 | CDP-4-dehydro-6-deoxyglucose reductase inhibitor |
| 0,828 | 0,005 | Antiinfective |
| 0,827 | 0,004 | DNA-(apurinic or apyrimidinic site) lyase inhibitor |
| 0,835 | 0,013 | Antiseborrheic |
| 0,824 | 0,002 | Mannan endo-1,6-alpha-mannosidase inhibitor |
| 0,824 | 0,002 | D-alanine 2-hydroxymethyltransferase inhibitor |
| 0,823 | 0,004 | UGT1A6 substrate |
| 0,821 | 0,002 | 3-Oxoadipate enol-lactonase inhibitor |
| 0,821 | 0,003 | Thymidylate 5'-phosphatase inhibitor |
| 0,823 | 0,004 | Fibrinolytic |
| 0,822 | 0,004 | Centromere associated protein inhibitor |
| 0,819 | 0,002 | 3-Hydroxyphenylacetate 6-hydroxylase inhibitor |
| 0,821 | 0,003 | Catechol oxidase inhibitor |
| 0,819 | 0,002 | GABA C receptor agonist |
| 0,817 | 0,002 | Diiodophenylpyruvate reductase inhibitor |
| 0,816 | 0,001 | Glycerol-1-phosphatase inhibitor |
| 0,814 | 0,002 | Glycerol dehydrogenase (NADP+) inhibitor |
| 0,814 | 0,002 | Glycerol 2-dehydrogenase (NADP+) inhibitor |
| 0,813 | 0,002 | N-acetyl-gamma-glutamyl-phosphate reductase inhibitor |
| 0,814 | 0,003 | Phenylacetate-CoA ligase inhibitor |
| 0,814 | 0,003 | (R)-Pantolactone dehydrogenase (flavin) inhibitor |
| 0,814 | 0,003 | (R)-6-hydroxynicotine oxidase inhibitor |
| 0,814 | 0,003 | Bothrolysin inhibitor |
| 0,812 | 0,002 | N-hydroxy-2-acetamidofluorene reductase inhibitor |
| 0,812 | 0,002 | Benzaldehyde dehydrogenase (NADP+) inhibitor |
| 0,812 | 0,002 | Trans-pentaprenyltranstransferase inhibitor |
| 0,811 | 0,002 | Pyruvate dehydrogenase (cytochrome) inhibitor |
| 0,811 | 0,002 | Trans-2-enoyl-CoA reductase (NAD+) inhibitor |
| 0,811 | 0,004 | Histidine N-acetyltransferase inhibitor |
| 0,811 | 0,004 | D-lactaldehyde dehydrogenase inhibitor |
| 0,815 | 0,008 | Membrane permeability inhibitor |
| 0,809 | 0,003 | Inulinase inhibitor |
| 0,809 | 0,003 | Alkenylglycerophosphoethanolamine hydrolase inhibitor |
| 0,809 | 0,003 | 4-Phytase inhibitor |
| 0,808 | 0,002 | Glycolate dehydrogenase inhibitor |
| 0,807 | 0,004 | Aryl-acylamidase inhibitor |
| 0,804 | 0,003 | Glycopeptide alpha-N-acetylgalactosaminidase inhibitor |
| 0,803 | 0,003 | Acetylornithine deacetylase inhibitor |
| 0,803 | 0,004 | P-benzoquinone reductase (NADPH) inhibitor |
| 0,814 | 0,015 | Mucomembranous protector |
| 0,801 | 0,002 | 2,4-Diaminopentanoate dehydrogenase inhibitor |
| 0,801 | 0,002 | 3-Hydroxybutyryl-CoA dehydrogenase inhibitor |
| 0,801 | 0,002 | Lysine 6-dehydrogenase inhibitor |
| 0,801 | 0,002 | tRNA nucleotidyltransferase inhibitor |
| 0,801 | 0,002 | Pantoate 4-dehydrogenase inhibitor |
| 0,801 | 0,002 | Aspartyl aminopeptidase inhibitor |
| 0,801 | 0,003 | 4-Hydroxyphenylacetate 3-monooxygenase inhibitor |
| 0,797 | 0,002 | Nicotine dehydrogenase inhibitor |
| 0,798 | 0,004 | Phosphoinositide 5-phosphatase inhibitor |
| 0,796 | 0,002 | Quinoprotein glucose dehydrogenase inhibitor |
| 0,795 | 0,003 | Polygalacturonase inhibitor |
| 0,794 | 0,003 | Cyclomaltodextrinase inhibitor |
| 0,793 | 0,003 | N-Acyl-D-amino-acid deacylase inhibitor |
| 0,793 | 0,003 | L-threonine 3-dehydrogenase inhibitor |
| 0,790 | 0,002 | Phenol 2-monooxygenase inhibitor |
| 0,788 | 0,001 | 3,4-Dihydroxy-9,10-secoandrosta-1,3,5(10)-triene-9,17-dione 4,5-dioxygenase inhibitor |
| 0,789 | 0,002 | Cystathionine beta-synthase inhibitor |
| 0,805 | 0,020 | CYP2J substrate |
| 0,795 | 0,010 | Lysase inhibitor |
| 0,798 | 0,014 | Mucositis treatment |
| 0,787 | 0,004 | Phosphatidylglycerophosphatase inhibitor |
| 0,785 | 0,004 | Isopenicillin-N epimerase inhibitor |
| 0,785 | 0,004 | Sulfite dehydrogenase inhibitor |
| 0,784 | 0,003 | Phenylalanine(histidine) transaminase inhibitor |
| 0,783 | 0,002 | Methylaspartate ammonia-lyase inhibitor |
| 0,784 | 0,003 | Nitrilase inhibitor |
| 0,783 | 0,003 | Arylsulfatase inhibitor |
| 0,784 | 0,003 | Nitrite reductase [NAD(P)H] inhibitor |
| 0,782 | 0,002 | Diphosphomevalonate decarboxylase inhibitor |
| 0,781 | 0,003 | Hydroxylamine reductase (NADH) inhibitor |
| 0,780 | 0,002 | 3-Demethylubiquinone-9 3-O-methyltransferase inhibitor |
| 0,782 | 0,005 | Methylumbelliferyl-acetate deacetylase inhibitor |
| 0,786 | 0,009 | JAK2 expression inhibitor |
| 0,779 | 0,003 | Protein-Npi-phosphohistidine-sugar phosphotransferase inhibitor |
| 0,779 | 0,004 | Kidney function stimulant |
| 0,789 | 0,014 | Beta-adrenergic receptor kinase inhibitor |
| 0,789 | 0,014 | G-protein-coupled receptor kinase inhibitor |
| 0,777 | 0,004 | APOA1 expression enhancer |
| 0,776 | 0,003 | 2,3-Dihydroxyindole 2,3-dioxygenase inhibitor |
| 0,780 | 0,007 | Linoleate diol synthase inhibitor |
| 0,774 | 0,002 | Ferredoxin-nitrite reductase inhibitor |
| 0,773 | 0,002 | Hippurate hydrolase inhibitor |
| 0,774 | 0,002 | Beta-amylase inhibitor |
| 0,779 | 0,009 | Carboxypeptidase Taq inhibitor |
| 0,772 | 0,002 | Carboxylate reductase inhibitor |
| 0,785 | 0,016 | Glycosylphosphatidylinositol phospholipase D inhibitor |
| 0,779 | 0,011 | Prostaglandin-E2 9-reductase inhibitor |
| 0,774 | 0,006 | Lipoprotein lipase inhibitor |
| 0,769 | 0,002 | D-amino-acid dehydrogenase inhibitor |
| 0,782 | 0,016 | CYP2J2 substrate |
| 0,767 | 0,001 | Quinate 5-dehydrogenase inhibitor |
| 0,768 | 0,002 | Succinate-semialdehyde dehydrogenase [NAD(P)+] inhibitor |
| 0,768 | 0,003 | Peptide-tryptophan 2,3-dioxygenase inhibitor |
| 0,766 | 0,003 | Chitosanase inhibitor |
| 0,766 | 0,003 | Pyruvate dehydrogenase (lipoamide) inhibitor |
| 0,765 | 0,002 | Aminomuconate-semialdehyde dehydrogenase inhibitor |
| 0,765 | 0,003 | 2-Enoate reductase inhibitor |
| 0,764 | 0,002 | Phosphoglycerate mutase inhibitor |
| 0,762 | 0,002 | 2,4-Dichlorophenol 6-monooxygenase inhibitor |
| 0,763 | 0,003 | Licheninase inhibitor |
| 0,760 | 0,001 | Tannase inhibitor |
| 0,762 | 0,003 | Dimethylmaleate hydratase inhibitor |
| 0,761 | 0,002 | Glucan endo-1,3-alpha-glucosidase inhibitor |
| 0,762 | 0,003 | Arylesterase inhibitor |
| 0,759 | 0,002 | Chloramphenicol O-acetyltransferase inhibitor |
| 0,761 | 0,007 | Manganese peroxidase inhibitor |
| 0,755 | 0,001 | Orotate reductase (NADPH) inhibitor |
| 0,754 | 0,002 | 4-Hydroxybenzoate 3-monooxygenase inhibitor |
| 0,754 | 0,001 | 3,4-Dihydroxyphenylacetate 2,3-dioxygenase inhibitor |
| 0,754 | 0,003 | H+-exporting ATPase inhibitor |
| 0,753 | 0,003 | Cyanoalanine nitrilase inhibitor |
| 0,752 | 0,002 | 3-Aminobutyryl-CoA ammonia-lyase inhibitor |
| 0,758 | 0,008 | Phthalate 4,5-dioxygenase inhibitor |
| 0,760 | 0,011 | Complement factor D inhibitor |
| 0,750 | 0,002 | L-iduronidase inhibitor |
| 0,751 | 0,003 | Myosin ATPase inhibitor |
| 0,750 | 0,002 | L-amino-acid oxidase inhibitor |
| 0,751 | 0,003 | Alpha-N-acetylglucosaminidase inhibitor |
| 0,751 | 0,004 | Beta-mannosidase inhibitor |
| 0,750 | 0,003 | Glutaminase inhibitor |
| 0,750 | 0,003 | Thiosulfate dehydrogenase inhibitor |
| 0,749 | 0,003 | 1-Alkylglycerophosphocholine O-acetyltransferase inhibitor |
| 0,748 | 0,003 | Plasmanylethanolamine desaturase inhibitor |
| 0,748 | 0,004 | Alcohol dehydrogenase (NADP+) inhibitor |
| 0,752 | 0,008 | Glyceryl-ether monooxygenase inhibitor |
| 0,747 | 0,003 | Benzoylformate decarboxylase inhibitor |
| 0,748 | 0,005 | Insulysin inhibitor |
| 0,770 | 0,027 | Acrocylindropepsin inhibitor |
| 0,770 | 0,027 | Chymosin inhibitor |
| 0,770 | 0,027 | Saccharopepsin inhibitor |
| 0,746 | 0,004 | UGT2B1 substrate |
| 0,743 | 0,002 | Cyclamate sulfohydrolase inhibitor |
| 0,743 | 0,003 | Acetate kinase inhibitor |
| 0,743 | 0,004 | Leukotriene-B4 20-monooxygenase inhibitor |
| 0,741 | 0,003 | Gly-X carboxypeptidase inhibitor |
| 0,779 | 0,042 | Phobic disorders treatment |
| 0,741 | 0,004 | Biotinidase inhibitor |
| 0,740 | 0,005 | X-methyl-His dipeptidase inhibitor |
| 0,737 | 0,002 | Vomilenine glucosyltransferase inhibitor |
| 0,739 | 0,004 | N-hydroxyarylamine O-acetyltransferase inhibitor |
| 0,737 | 0,002 | Leucine dehydrogenase inhibitor |
| 0,737 | 0,004 | Coccolysin inhibitor |
| 0,734 | 0,003 | 4-Coumarate-CoA ligase inhibitor |
| 0,733 | 0,003 | Phenylalanine 4-hydroxylase inhibitor |
| 0,735 | 0,005 | Butyrate-CoA ligase inhibitor |
| 0,733 | 0,003 | Nicotinamidase inhibitor |
| 0,733 | 0,004 | Procollagen N-endopeptidase inhibitor |
| 0,731 | 0,002 | Dopachrome isomerase inhibitor |
| 0,730 | 0,003 | Carnosine synthase inhibitor |
| 0,732 | 0,005 | HMOX1 expression enhancer |
| 0,736 | 0,010 | Pitrilysin inhibitor |
| 0,728 | 0,003 | 2,2-Dialkylglycine decarboxylase (pyruvate) inhibitor |
| 0,727 | 0,003 | Cyclopentanone monooxygenase inhibitor |
| 0,726 | 0,002 | Urease inhibitor |
| 0,729 | 0,006 | Ecdysone 20-monooxygenase inhibitor |
| 0,727 | 0,004 | Rubredoxin-NAD+ reductase inhibitor |
| 0,725 | 0,002 | Lombricine kinase inhibitor |
| 0,741 | 0,018 | Protein-glutamate methylesterase inhibitor |
| 0,725 | 0,002 | Dihydroxy-acid dehydratase inhibitor |
| 0,744 | 0,022 | Nicotinic alpha6beta3beta4alpha5 receptor antagonist |
| 0,727 | 0,007 | Lysostaphin inhibitor |
| 0,723 | 0,002 | 3-Cyanoalanine hydratase inhibitor |
| 0,721 | 0,003 | Aryl-alcohol dehydrogenase inhibitor |
| 0,718 | 0,001 | 4-Hydroxybenzoate decarboxylase inhibitor |
| 0,718 | 0,001 | 4,5-Dihydroxyphthalate decarboxylase inhibitor |
| 0,733 | 0,016 | Alkylacetylglycerophosphatase inhibitor |
| 0,737 | 0,021 | Acylcarnitine hydrolase inhibitor |
| 0,719 | 0,004 | N-(long-chain-acyl)ethanolamine deacylase inhibitor |
| 0,719 | 0,004 | Malate dehydrogenase (acceptor) inhibitor |
| 0,717 | 0,003 | DNA-3-methyladenine glycosylase I inhibitor |
| 0,718 | 0,005 | Cyclohexanone monooxygenase inhibitor |
| 0,717 | 0,004 | Monophenol monooxygenase inhibitor |
| 0,715 | 0,003 | Porphobilinogen synthase inhibitor |
| 0,715 | 0,003 | Gallate decarboxylase inhibitor |
| 0,713 | 0,002 | Protocatechuate 3,4-dioxygenase inhibitor |
| 0,713 | 0,003 | Indoleacetaldoxime dehydratase inhibitor |
| 0,710 | 0,002 | Acyl-lysine deacylase inhibitor |
| 0,709 | 0,006 | Aspartyltransferase inhibitor |
| 0,717 | 0,013 | Oxidoreductase inhibitor |
| 0,707 | 0,004 | Aldehyde dehydrogenase (NADP+) inhibitor |
| 0,703 | 0,002 | Glutathione dehydrogenase (ascorbate) inhibitor |
| 0,706 | 0,005 | Cytochrome-b5 reductase inhibitor |
| 0,708 | 0,008 | UGT1A9 substrate |
| 0,702 | 0,003 | Alanine transaminase inhibitor |
| 0,712 | 0,014 | UDP-glucuronosyltransferase substrate |
| 0,700 | 0,002 | (S)-3-hydroxyacid ester dehydrogenase inhibitor |
| 0,700 | 0,002 | Prephenate dehydrogenase inhibitor |
| 0,700 | 0,003 | Sphinganine-1-phosphate aldolase inhibitor |
| 0,700 | 0,003 | Glucuronate isomerase inhibitor |
| 0,700 | 0,004 | Erythropoiesis stimulant |
| 0,718 | 0,023 | TP53 expression enhancer |
